# The Role of Multidetector Computed Tomography in the Early Diagnosis of Invasive Pulmonary Aspergillosis in Patients with Febrile Neutropenia Undergoing Hematopoietic Stem Cell Transplantation

**DOI:** 10.5152/tjh.2011.78

**Published:** 2012-03-05

**Authors:** Nazan Çiledağ, Kemal Arda, Bilgin Kadri Arıbaş, Ali Irfan Emre Tekgündüz, Fevzi Altuntaş

**Affiliations:** 1 Ankara Oncology Education and Research Hospital, Department of Radiology, Ankara, Turkey; 2 Ankara Oncology Education and Research Hospital, Department of Hematology and BMT Unit, Ankara, Turkey

**Keywords:** Multidetector computed tomography, Invasive pulmonary aspergillosis, Febrile neutropenia, Hematopoietic stem cell transplantation

## Abstract

**Objective:** To evaluate vessel involvement and the role of multidetector computed tomography (MDCT) in the earlydiagnosis of invasive pulmonary aspergillosis (IPA) in patients with febrile neutropenia and antibiotic-resistant feverundergoing autologous bone morrow transplantation.

**Material and Methods:** In all, 74 pulmonary MDCT examinations in 37 consecutive hematopoietic stem celltransplantation patients with febrile neutropenia and clinically suspected IPA were retrospectively evaluated.

**Results:** Diagnosis of IPA was based on Fungal Infections Cooperative Group, and National Institute of Allergy andInfectious Diseases Mycoses Study Consensus Group criteria. In all, 0, 14, and 11 patients were diagnosed as proven,probable, and possible IPA, respectively. Among the 25 patients accepted as probable and possible IPA, all had pulmonaryMDCT findings consistent with IPA. The remaining 12 patients were accepted as having fever of unknown origin (FUO)and had patent vessels based on MDCT findings.In the patients with probable and possible IPA, 72 focal pulmonary lesions were observed; in 41 of the 72 (57%) lesionsvascular occlusion was noted and the CT halo sign was observed in 25 of these 41 (61%) lesions. Resolution of feveroccurred following antifungal therapy in 19 (76%) of the 25 patients with probable and possible IPA. In all, 6 (25%)of the patients diagnosed as IPA died during follow-up. Transplant-related mortality 100 d post transplant in patientswith IPA and FUO was 24% and 0%, respectively.

**Conclusion:** In conclusion, MDCT has a potential role in the early diagnosis of IPA via detection of vessel occlusion.

## Abstract (tr)

**Amaç:** Otolog kemik iliği transplantasyonlu febril nötropenik ve antibiyotiğe dirençli orjini bilinmeyen hastalarda, vasküler tutulum ve invaziv pulmoner aspergillozis (İPA) erken tanısında multidedektör bilgisayarlı tomografi (MDBT)’nin rolünü değerlendirmek amaçlanmıştır.

**Gereç ve Yöntemler:** Orjini bilinmeyen geniş spektrumlu antibiyotiğe dirençli ateşi olan, İPA şüphesi bulunan 37 olgunun 74 pulmoner MDBT incelemesi retrospektif değerlendirildi.

**Bulgular:** İnvaziv pulmoner aspergillozis tanısı mikrobiyolojik, radyolojik bulgularla birlikte host faktörleri değerlendirilerek konuldu. 25 olguya EORTC/MSG kriterlerine göre sırasıyla 0, 14, 11 olguya kanıtlanmış, olası, muhtemel İPA tanısı konuldu. Olası veya muhtemel İPA tanısı konan 25 olgunun MDBT’sinde İPA ile uyumlu bulgular görüldü. Ne kültürde üreme, ne de radyolojik pozitif bulgu saptanmayan 12 olguda (nedeni bilinmeyen ateş) MDBT’de vasküler yapılar patent idi. Olası/muhtemel İPA tanısı konan 25 olguda total 72 fokal pulmoner lezyon saptandı. 72 lezyonun 41’inde (%57) MDBT’de İPA’yı destekleyen vasküler oklüzyon saptandı. Olası/muhtemel İPA tanısı konan 25 olgunun 19’unda (%76) klinik düzelme, ateşin düşüşü gözlendi. İnvaziv pulmoner aspergillozis tanısı konan 6 olgu takipler sırasında kaybedildi. İnvaziv pulmoner aspergillozis ve nedeni binmeyen ateş saptanan olgularda 100 günlük takip sonrasında transplant ilişkili mortalite oranı sırasıyla %24 ve %0 idi.

**Sonuç:** Sonuç olarak, vasküler tutulumu gösterebilmesi nedeniyle MDBT’nin İPA tanısında potansiyel rolü olduğu

kanısına varıldı.

## INTRODUCTION

Fungal infection is one of the primary causes of posthematopoietic stem cell transplantation (HSCT) mortalityand morbidity, despite routine prophylaxis for commonpathogenic organisms and empiric treatment of febrileepisodes during the early neutropenic period [1,2]. In theera of routine fluconazole prophylaxis for high-risk populations,molds-especially Aspergillus species-became themost common and life-threatening pathogenic organism,leading to fungal pneumonia in febrile neutropenic patientsand causing 90% of such infections [3,4]. Early and accuratediagnosis of invasive pulmonary aspergillosis (IPA) inimmunocompromised patients is the ultimate method oflowering the mortality and morbidity rates associated withthe pathogen [5]. A 10-d delay in initiation of antifungaltherapy nearly doubles the IPA-related mortality rate [5].

High-resolution CT visualization of the halo sign-indicativeof a hemorrhagic pulmonary nodule-is an early signof IPA with high sensitivity and specificity [6]; however,differential diagnosis of the halo sign is quite complex,as it may be observed as a result of infection with othermicroorganisms [(Candida spp., mucormycosis, cryptococcosis,cytomegalovirus (CMV ), herpes simplex virus(HSV)] and other diseases, including Wegener granulomatosis,pulmonary metastases of hypervascular tumors, andKaposi’s sarcoma [7,8]. Pathologic examination of noduleswith the halo sign showed that the halo around a central fungal lesion corresponded to a nodule surrounded by arim of coagulation necrosis due to the vascular invasionthat causes thrombotic occlusion and ischemic necrosis[9]. Due to limitations of these radiological findings, afew recent studies evaluated the role of multidetectorcomputed tomographic (MDCT) angiography in the earlyand accurate diagnosis of IPA [10].

The primary aim of the present study was to evaluatevessel involvement and the role of multidetector computedtomography (MDCT) in the early diagnosis of invasive IPAin patients with febrile neutropenia and antibiotic-resistantfever undergoing HSCT.

## MATERIALS AND METHODS

In all, 37 consecutive HSCT patients (24 male and 13female) with a mean age of 41 ± 19 years (range: 4-68years) and clinically suspected IPA due to febrile neutropeniaand broad-spectrum antibiotic-resistant fever ofunknown origin that underwent 74 pulmonary MDCTexaminations between March 2009 and June 2010 wereretrospectively evaluated. In total, 27 of the patients(73%) had acute leukemia, 2 (5%) had chronic leukemia,7 (19%) had lymphoma, and 1 (3%) had multiplemyeloma. The patients with acute and chronic leukemiawere treated with allogeneic HSCT from 6/6 HLA-matchedrelated donors. The other patients underwent autologousperipheral stem cell transplantation and were neutropenic (absolute neutrophil count <500 mm-3 for at least 11 d)following myeloablative conditioning. The conditioningregimen in the patients with acute/chronic leukemia wasTBI/Cy or Bu/Cy, whereas patients with lymphoma andmyeloma received BEAM and MEL200, respectively.

All the HSCT patients received levofloxacin, fluconazole,acyclovir, and trimethoprim-sulfamethoxazole asprimary prophylaxis. Allogeneic HSCT recipients receivedcyclosporin and short-term methotrexate for prophylaxisof graft versus host disease. According to our hematology/stem cell transplantation protocol, febrile neutropenicpatients that were unresponsive to broad-spectrum antibiotics(imipenem or meropenem) for 72 h were evaluatedfor opportunistic fungal pathogens. We adapteda similar (not the same) pre-emptive strategy pioneeredby Maertens et al. [11[—serum galactomannan (GM )assay (twice weekly) and thoracic MDCT. Patients thathad hemodynamic instability and/or 2 consecutive positiveserum GM assays (ELISA: optical density ≥0.5), and/or thoracic MDCT findings suggesting IPA (pulmonarynodule irrespective of the halo sign, air crescent sign, orcavitation) supported by mycological evidence of molds(presence of fungal elements indicating molds in sputum/bronchoalveolar lavage fluid or positive culture for molds)were treated with antifungal agents. Patients with mycologicalevidence of Aspergillus spp. received voriconazole;all others were treated with caspofungin.

Persistent or relapsing fever was not considered anindication for antifungal therapy. Once initiated, antifungaltherapy was continued for 6 weeks. In patients thatwere unresponsive to pre-emptive antifungal treatment,MDCT was repeated; hence, it was performed more thanonce in some patients. Patients with renal insufficiencywere excluded from the study. The study protocol wasapproved by Ankara oncology hospital review board andwritten informed consent was received from all patients.The diagnosis of IPA was based on evaluation of hostfactors, together with mycological and radiological findings,according to the international consensus guidelines[12]. Due to safety concerns, no patient underwent transbronchial/percutaneous lung biopsy or needle aspirationof radiologically suspected lesions, allowing histopathologicor cytopathologic examination.Pulmonary CT was performed caudocranially withan MDCT scanner (GE Medical Systems, Milwaukee,WI, USA) after administration of 100 mL of intravenouscontrast media (ioversol, 350 mg mL-1 organically boundiodine, Optiray, Covidien, Tyco, USA) at a rate of 4 mL s-1.MDCT was performed with 20-s delay during breath-hold.Scanning parameters were as follows: slice thickness: 1.25mm; helical rotation time: 0.5 s; collimation: 1.25 mm;voltage: 120 kV; current: 120 mAs. After completion ofCT scanning the reconstruction process was applied andmultiplanar reconstructed (sagittal, coronal, and obliqueplanes) images were created. Axial images were evaluatedfor nodules, cavitation, and the halo sign, and thenany focal pulmonary lesions observed were evaluated forvascular involvement defined as an interruption of thevessel at the border of the focal nodular lesion in multiplanarreconstructed images—and evaluated based on theconsensus of 2 radiologists. Results were correlated withclinical follow-up data, including serum galactomannanvalues and CT findings during a median follow-up of 93 d(range: 10-220 d).

## STATISTICAL ANALYSIS

Statistical analysis was performed using SPSS v.13.0.Categorical variables are presented as numbers andpercentages, and continuous variables are presented asmean ± SD or median.

## RESULTS

**Figure 1:**
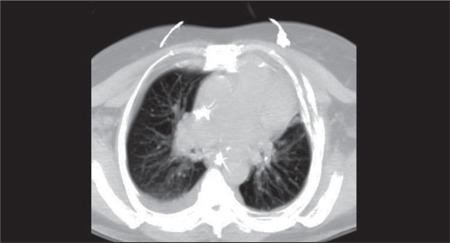
Contrast-enhanced MDCT axial plane maximumintensity projection image shows patent vessels.

According to EORTC/MSG criteria, 0, 14, and 11patients were diagnosed as proven, probable, and possibleIPA, respectively. Sputum culture in 2 patients showedinfection with Aspergillus spp. Among the 14 patientswith probable IPA, 2 had Aspergillus-positive culture and12 patients had 2 consecutive positive GM assays. These14 patients also had pulmonary CT findings indicative ofIPA. In all, 11 of the 37 patients that had both host andradiological findings suggesting IPA did not have cultureor non-culture-based test results consistent with IPA(possible IPA). In the remaining 12 patients the diagnosisof IPA was excluded by any level of evidence and thesepatients were regarded as having fever of unknown origin (FUO). All the patients with FUO had patent vessels, basedon MDCT (Figure 1). Among the patients with FUO, 3had consolidation, with strong and patent vessels in andaround the consolidation, whereas the other 9 patientshad no pathology based on MDCT. On the other hand,18 (72%) of the patients that were diagnosed as probableand possible IPA had abnormal MDCT findings (Table 1).

**Figure 2:**
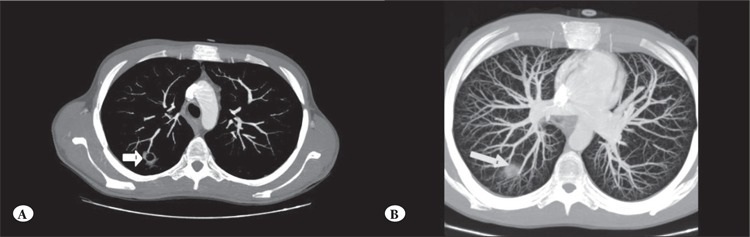
A 24-year-old male with acute myeloid leukemia. (A) Contrast-enhanced MDCT scan of the chest obtained with lungwindow settings shows a cavitary nodular lesion (arrow) with a peripheral halo sign. (B) Contrast-enhanced MDCT axial planemaximum intensity projection image shows interruption of the peripheral segmental pulmonary artery (arrow) at level of the pulmonarynodule.

In the patients with probable and possible IPA 72 focal pulmonary lesions were detected via MDCT; the lesionsranged from 6 to 40 mm (mean: 1.7 ± 0.6 mm). In 41 ofthe 72 (57%) lesions vascular occlusion was noted, whichwas defined as an interruption of a vessel at the border of afocal lesion (without depiction of the vessel) or peripheralto the lesion (Figure 2A,B). The CT halo sign was noted in25 of the 41 (61%) lesions. In total, 9 cavitary pulmonarynodules with a surrounding halo of ground-glass attenuationwere detected using MDCT. Clinical improvementand resolution of fever was achieved following antifungaltherapy in 19 (76%) of the 25 patients with probable andpossible IPA. In patients with clinical deterioration, therewas also progression of radiological findings based on MDCT. In all, 6 (25%) patients that were diagnosed asIPA died during follow-up. In 3 of the 12 patients withnormal MDCT findings and persistent fever MDTC wasrepeated and the results were again normal; there were noclinical or laboratory findings supporting the diagnosis ofIPA among them. Transplant-related mortality (TRM ) 100d post transplant in the patients with IPA and FUO was24% and 0%, respectively.

## DISCUSSION

IPA is one of the most serious pulmonary complicationsof HSCT. The incidence of IPA varies widely according tostudy population. The risk of proven and probable IPAamong autologous transplant recipients is quite low (0.5%-1%), as compared to patients undergoing allogeneic HSCTor those treated with induction chemotherapy for acuteleukemia (3%-7%) [13-15]. Although rare, IPA is associatedwith high mortality and morbidity rates following HSCT,especially during the early neutropenic period [10,16]. Themortality rate due to IPA when treated with appropriateantifungal therapy ranges from 30% to 80% [17]. Early and accurate diagnosis of IPA is critical because initiationof specific antifungal therapy decreases the mortality ratefrom 80% to 30% [6]. In the present study MDCT findingsin 37 HSCT patients with neutropenia and antibioticresistantFUO, and clinically suspected IPA were retrospectivelyevaluated via in order to detect the existence of focalpulmonary lesions, the halo sign, and vascular occlusion.Due to severe thrombocytopenia, poor clinical condition,and high risk of complications associated with invasivediagnostic procedures, diagnoses were based on clinical,microbiological, and radiological findings.

A pulmonary nodule with the halo sign observed withhigh-resolution CT is an important and early sign of IPA;however, the halo sign is a non-specific imaging findingthat may be observed in other diseases [7,8]. During thelast 10 years technological progress in medical imaging hasresulted in MDCT, which has many advantages over singledetectorhelical CT, including shorter acquisition time,greater anatomic coverage, and superior image resolutionduring a single breath-hold, facilitating improved patientcomfort and excellent three dimensional (3D) reconstructions.All these factors substantially increase diagnosticaccuracy via state-of-the-art image quality. 3D isotropicvolume imaging is possible with MDCT and providesexcellent anatomical imaging of the thorax, thereby significantlyincreasing the diagnostic yield. MDCT has resultedin a paradigm shift in vascular imaging—from conventionalcatheter angiography to MDCT angiography—asMDCT provides image quality that equals or surpassesthat of conventional angiography.

Recent advances in 3D volume rendering enable detectionof occlusion of peripheral vessels at the border of asuspicious fungal lesion. Hypothetically, vasculitic changein peripheral vessels can cause occlusion of peripheralvessels. Vessel occlusion secondary to vascular invasion byIPA results in edema, which is referred to as the CT-halosign. Sonnet el al. reported that direct detection of vesselocclusion, which was defined as an interruption of a vesselat the border of a focal nodular pulmonary lesion, is anearlier finding than the halo sign and may be useful in theearly diagnoses of IPA [10]. In the present study only 61%of the patients with vascular occlusion around the pulmonarynodules had the halo sign, which is in agreementwith the findings of Sonnet el al. [10], and therefore itseems logical to conclude that MDCT could be a valuabletool for the early diagnosis of IPA. As the present study’scohort consisted of only 37 patients, it is impossible todefinitively state the value of MDCT in the diagnosis ofIPA based on the present findings.

According to the recently published EORTC/MSGcriteria, the rate of proven/probable IPA in the presentstudy was 38%, which is much higher than expected, andthis discrepancy has several possible causes. In order tofocus on a population with a high risk for IPA we includedonly patients with defined host factors based on EORTC/MSG criteria. In addition to the allogeneic transplantrecipients, all 8 patients that were treated with autologousHSCT had profound neutropenia for at least 11 d.Autologous HSCT patients that did not fulfill EORTC/MSG host criteria were not included in the study. Additionally,there are some drawbacks associated with the useof serum GM -ELISA assay for the detection of IPA. Recentdata show that the positive predictive value (58%-73%)of the test is not optimal [15]. In order to increase thesensitivity and safety of the pre-emptive approach we useda rather low threshold for positivity (optical density ≥0.5).Moreover, a vast majority of the patients (n = 29 [78%])were treated with a conditioning regimen that includedhigh-dose cyclophosphamide, which can cause false positiveGM assay results [18]. In consideration of the fact thatnone of the present study’s patients had proven IPA andall 14 patients with probable IPA had 2 consecutive positiveGM assays, the high rate of IPA observed may be anoverestimation of the real picture.

The diagnosis of IPA in the present study was basedon evaluation of host factors, together with mycologicaland radiological findings, according to the internationalconsensus guidelines [12]. The major limitation of thepresent study is the lack of histopathologic diagnosis ofIPA, which was not performed due to safety concerns. Inconclusion, MDCT shows promise as an imaging techniquefor accurate and early detection of vessel occlusionin HSCT patients with suspected IPA; however, additionalresearch with larger patient populations is needed tofurther establish its accuracy.

## CONFLICT OF INTEREST STATEMENT

The authors of this paper have no conflicts of interest,including specific financial interests, relationships, and/or affiliations relevant to the subject matter or materials included.

## Figures and Tables

**Table 1:**
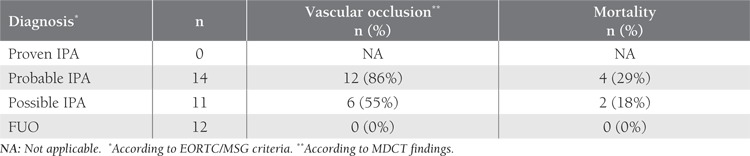
MDCt Findings and Clinical Outcomes in the Study Group
